# Metformin and Biliary Tract Cancer in Patients With Type 2 Diabetes

**DOI:** 10.3389/fonc.2020.587666

**Published:** 2020-10-27

**Authors:** Chin-Hsiao Tseng

**Affiliations:** ^1^ Department of Internal Medicine, National Taiwan University College of Medicine, Taipei, Taiwan; ^2^ Division of Endocrinology and Metabolism, Department of Internal Medicine, National Taiwan University Hospital, Taipei, Taiwan; ^3^ Division of Environmental Health and Occupational Medicine of the National Health Research Institutes, Zhunan, Taiwan

**Keywords:** biliary tract cancer, diabetes mellitus, metformin, Taiwan, survival

## Abstract

**Aim:**

This retrospective cohort study evaluated whether metformin use in patients with type 2 diabetes mellitus might reduce the risk of biliary tract cancer (BTC); and explored whether metformin use might affect the overall survival in patients who developed BTC.

**Methods:**

New-onset type 2 diabetes patients aged 25–75 years during 1999–2005 were enrolled from the Taiwan’s National Health Insurance and followed up until December 31, 2011. A total of 287,995 ever users and 16,229 never users were identified (unmatched original cohort) and a 1:1 matched pairs of 16,229 ever users and 16,229 never users based on propensity score (PS) were created (matched cohort). Hazard ratios were estimated by three Cox regression models: 1) adjusted for PS; 2) incorporated with the inverse probability of treatment weighting using PS; and 3) all covariates treated as independent variables. Overall survival was compared between ever users and never users of metformin who developed BTC.

**Results:**

In the unmatched cohort, 73 never users and 523 ever users developed BTC, with respective incidence of 100.36 and 38.06 per 100,000 person-years. An overall risk reduction was observed in metformin users in all three regression models with respective hazard ratio (95% confidence interval) of 0.442 (0.344-0.568), 0.377 (0.295-0.481), and 0.477 (0.370-0.615). The tertile analyses showed a dose-response pattern with a neutral effect in the first tertile when metformin use was <2 years and a significant risk reduction in the second and third tertiles. Findings in the matched cohort were consistent with those observed in the unmatched cohort. The overall survival did not differ significantly between ever and never users of metformin among patients who developed BTC.

**Conclusions:**

Metformin significantly reduces the overall risk of BTC by 50%–60%. A dose-response effect is observed and users of approximately 2 years show significantly reduced risk. However, metformin does not affect the overall survival in patients with BTC.

## Introduction

Biliary tract cancer (BTC) arises from the epithelium of the biliary tree and can be classified as intrahepatic cholangiocarcinoma, extrahepatic cholangiocarcinoma, and gallbladder cancer according to its anatomical location. BTC is highly malignant and is always diagnosed at an advanced stage, with 5-year survival <10% for all subtypes (1, 2). Metabolism reprogramming with a shift from oxidative phosphorylation to glycolysis (Warburg effect or aerobic glycolysis) is a major feature of BTC (3).

Although the risk factors of BTC are not well characterized (3), patients with type 2 diabetes mellitus may have an increased risk of various types of cancer including BTC (4–7). Metformin is now considered the first-line therapy for type 2 diabetes mellitus because it exerts multiple beneficial effects beyond glycemic control, such as anti-inflammation, anti-atherosclerosis, anti-cancer and anti-aging (4). Despite a myriad of studies investigating the role of metformin use in diabetes patients in risk reduction of various cancers (8, 9), to our knowledge, only one previous clinic/hospital-based case-control study investigated the risk of BTC associated with metformin use (5). In this study the investigators estimated a 60% risk reduction with an odds ratio of 0.4 (95% confidence interval 0.2–0.9, *P*=0.04).

Because of the highly malignant nature of BTC, metformin does not provide a useful therapeutic benefit or improve the survival of the patients once BTC is diagnosed (10–12). However, in *in vitro* studies, metformin does inhibit the proliferation and viability of BTC and promote the apoptosis of BTC through various mechanisms. These may include the suppression of nuclear translocation of signal transducer and activator of transcription 3 and nuclear factor-kappa B through the activation of 5’-adenosine monophosphate-activated protein kinase (AMPK) (13), the inhibition of p-Akt and Bcl-2, probably through upregulation of the chloride intracellular channel 1 (14), or arrest of cell cycle by regulating the expression of Drosha-mediated multiple carcinogenic microRNAs (15). A recent *in vitro* study by Tang et al. suggested that metformin may alter cellular metabolism with the suppression of the Warburg effect by decreasing the expression of lactate dehydrogenase A in cholangiocarcinoma cell cultures (16). Furthermore, metformin may sensitize the anticancer effect of cisplatin on BTC through activating the oxidative stress-mediated mitochondrial cell death pathway (17). Therefore, even though metformin may not be useful as a therapeutic agent for BTC, its beneficial effect to prevent the early development of such a highly malignant cancer is worthy of investigation. The beneficial effect observed by Chaiteerakij et al. (5) should better be confirmed in different ethnicities with additional consideration of common methodological biases such as prevalent user bias, immortal time bias and confounding by indication.

The present study aimed at investigating whether metformin use might reduce the risk of BTC in Taiwanese patients with type 2 diabetes mellitus. Furthermore, we also explored whether the use of metformin might affect the overall survival in patients who developed BTC during follow-up.

## Materials and Methods

The Taiwan’s National Health Insurance (NHI) is a compulsory, universal and unique healthcare system that has been implemented since March 1995. It has a high coverage of over 99% of the whole population and has contracts with 93% of medical settings and with all in-hospitals nationwide. All disease diagnoses, prescribed medications and performed procedures are recorded as computerized database. The database can be used for academic research after approval by an ethics review board. The present study was approved with a number of 99274.

Disease diagnoses were coded by the International Classification of Diseases, Ninth Revision, Clinical Modification (ICD-9-CM) during the study period. Diabetes was coded 250.XX and BTC included 155.1 (malignant neoplasm of intrahepatic bile ducts) and 156 (malignant neoplasm of gallbladder and extrahepatic bile ducts).

**Figure 1:**
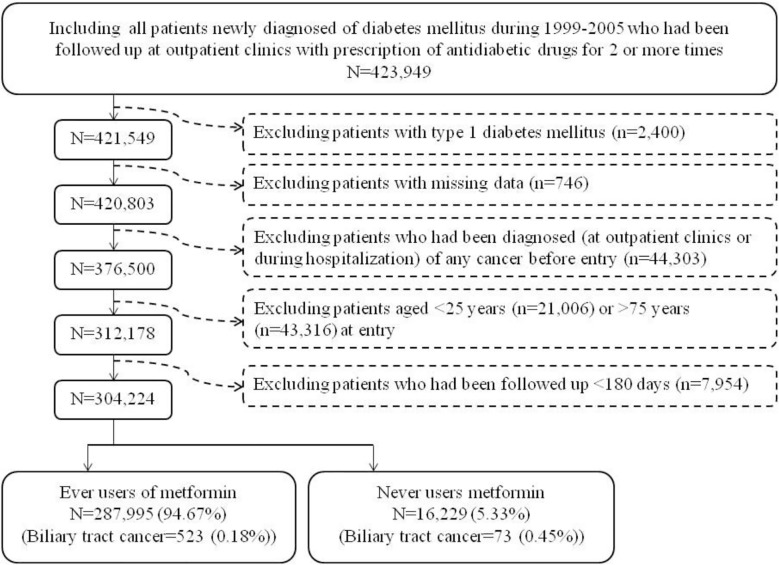
The procedures in creating the unmatched original cohort of ever and never users of metformin by using the reimbursement database of the National Health Insurance of Taiwan.

**Figure 1** shows the procedures followed in creating the unmatched original cohort used in the study. In brief, 423,949 patients with new-onset diabetes mellitus during 1999-2005 and ≥2 prescriptions of antidiabetic drugs in the outpatient clinics were first identified. After excluding patients with type 1 diabetes mellitus (n=2,400), missing data (n=746), with cancer diagnosis before entry (n=44,303), aged <25 years (n=21,006), aged >75 years (n=43,316), and with follow-up duration <180 days (n=7,954), a total of 304,224 patients were selected into the analyses. Among them, 287,995 patients had ever been prescribed metformin and 16,229 had never been treated with metformin (the unmatched original cohort). The Greedy 8→1 digit match algorithm was used to create a PS matched-pairs cohort (the matched cohort) of ever and never users according to the methods described by Parsons (18). These methods have also been used in our previous papers (19, 20). The PS was created by logistic regression with all the characteristics (collected until the end of follow-up) listed in **Table 1** and the date of entry treated as independent variables.

**Table 1:** Characteristics in metformin never users and ever users in the unmatched original cohort and in the propensity score matched cohort.

Variable	Unmatched original cohort								Matched cohort						
	Never users			Ever users					Never users		Ever users				
	(*n*=16229)			(*n*=287995)			*P* value	SD	*(n*=16229)		*(n*=16229)		*P* value	SD
	*n*	%		*n*	%				*n*	%	*n*	%			
**Demographic data**														
Age (years)*	63.63 ± 10.42			61.39 ± 10.22			<0.0001	-22.14	63.63 ± 10.42		63.90 ± 9.86		0.0150	3.49
Sex (Men)	9301	57.31		155141	53.87		<0.0001	-7.23	9301	57.31	9369	57.73	0.4451	0.58
**Occupation**														
I	6338	39.05		116164	40.34		<0.0001		6338	39.05	6403	39.45	0.8514	
II	3232	19.91		65956	22.90			7.63	3232	19.91	3179	19.59		-0.84
III	3409	21.01		56167	19.50			-3.75	3409	21.01	3409	21.01		0.21
IV	3250	20.03		49708	17.26			-7.57	3250	20.03	3238	19.95		-0.29
**Living region**														
Taipei	5461	33.65		97273	33.78		<0.0001		5461	33.65	5413	33.35	0.6716	
Northern	1658	10.22		34432	11.96			5.73	1658	10.22	1609	9.91		-1.05
Central	2838	17.49		51317	17.82			0.94	2838	17.49	2866	17.66		0.39
Southern	2807	17.30		46179	16.03			-3.51	2807	17.30	2785	17.16		-0.12
Kao-Ping and Eastern	3465	21.35		58794	20.41			-2.31	3465	21.35	3556	21.91		1.51
**Major comorbidities**														
Hypertension	13314	82.04		234768	81.52		0.0965	-1.41	13314	82.04	13318	82.06	0.9539	0.29
Dyslipidemia	11727	72.26		239093	83.02		<0.0001	27.63	11727	72.26	11702	72.11	0.7568	0.03
Obesity	440	2.71		16491	5.73		<0.0001	15.15	440	2.71	349	2.15	0.0010	-3.73
**Diabetes-related complications**														
Nephropathy	5665	34.91		77188	26.80		<0.0001	-19.03	5665	34.91	5606	34.54	0.4915	-1.48
Eye disease	3010	18.55		89040	30.92		<0.0001	29.38	3010	18.55	2784	17.15	0.0011	-3.88
Stroke	5403	33.29		83325	28.93		<0.0001	-10.00	5403	33.29	5396	33.25	0.9343	-0.08
Ischemic heart disease	7775	47.91		130776	45.41		<0.0001	-5.29	7775	47.91	7735	47.66	0.6567	-0.38
Peripheral arterial disease	3780	23.29		72131	25.05		<0.0001	4.24	3780	23.29	3776	23.27	0.9581	-0.24
**Antidiabetic drugs**														
Insulin	1350	8.32		6096	2.12		<0.0001	-29.93	1350	8.32	1009	6.22	<0.0001	-9.28
Sulfonylurea	11797	72.69		189774	65.89		<0.0001	-11.33	11797	72.69	12242	75.43	<0.0001	7.68
Meglitinide	1341	8.26		10347	3.59		<0.0001	-20.98	1341	8.26	1256	7.74	0.0820	-1.71
Acarbose	1834	11.30		14527	5.04		<0.0001	-22.51	1834	11.30	1809	11.15	0.6602	-1.10
Rosiglitazone	481	2.96		12955	4.50		<0.0001	8.55	481	2.96	490	3.02	0.7693	0.17
Pioglitazone	401	2.47		7013	2.44		0.7737	0.47	401	2.47	416	2.56	0.5951	0.41
**Potential risk factors of cancer**														
Chronic obstructive pulmonary disease	8093	49.87		140558	48.81		0.0085	-2.53	8093	49.87	8049	49.60	0.6252	-0.51
Tobacco abuse	459	2.83		11337	3.94		<0.0001	6.30	459	2.83	440	2.71	0.5205	-0.58
Alcohol-related diagnoses	1286	7.92		20192	7.01		<0.0001	-4.24	1286	7.92	1246	7.68	0.4077	-1.26
Gallstone	2261	13.93		36180	12.56		<0.0001	-4.53	2261	13.93	2274	14.01	0.8351	
History of Helicobacter pylori infection	5459	33.64		86596	30.07		<0.0001	-8.48	5459	33.64	5359	33.02	0.2390	-1.53
Epstein-Barr virus-related diagnoses	117	0.72		2057	0.71		0.9217	-0.12	117	0.72	108	0.67	0.5471	-0.70
Hepatitis B virus infection	730	4.50		12071	4.19		0.0583	-1.85	730	4.50	660	4.07	0.0550	-2.33
Hepatitis C virus infection	1058	6.52		14722	5.11		<0.0001	-6.55	1058	6.52	1010	6.22	0.2753	-1.44
Disease of pancreas	893	5.50		13702	4.76		<0.0001	-4.14	893	5.50	897	5.53	0.9225	-0.44
**Medications that are commonly used in diabetes patients or may affect cancer risk**														
Angiotensin converting enzyme inhibitor/angiotensin receptor blocker	11299		69.62	209234		72.65	<0.0001	6.86	11299	69.62	11210	69.07	0.2840	-1.13
Calcium channel blocker	10222		62.99	170516		59.21	<0.0001	-8.03	10222	62.99	10261	63.23	0.6537	0.64
Statin	8772		54.05	189124		65.67	<0.0001	24.98	8772	54.05	8704	53.63	0.4490	-0.62
Fibrate	5551		34.20	122878		42.67	<0.0001	18.21	5551	34.20	5527	34.06	0.7787	-0.18
Aspirin	9337		57.53	175631		60.98	<0.0001	7.17	9337	57.53	9238	56.92	0.2667	-0.94

Refer to Materials and Methods for the classification of occupation.

The specific drugs in the class of sulfonylurea include chlorpropamide, glibenclamide, glipizide, gliclazide, glimepiride and gliquidone.

*Age is expressed as mean ± standard deviation.

SD, standardized difference.

Cumulative duration of metformin therapy (months) was calculated and its tertiles were used to evaluate the dose-response relationship. Potential confounders were categorized into demographic data (age and sex), occupation, living region, major comorbidities (hypertension, dyslipidemia, and obesity), diabetes-related complications (nephropathy, eye disease, stroke, ischemic heart disease, and peripheral arterial disease), antidiabetic drugs (insulin, sulfonylurea, meglitinide, acarbose, rosiglitazone, and pioglitazone), potential risk factors of cancer (chronic obstructive pulmonary disease, tobacco abuse, alcohol-related diagnoses, gallstone, history of Helicobacter pylori infection, Epstein-Barr virus-related diagnoses, hepatitis B virus infection, hepatitis C virus infection, and disease of pancreas), and medications that are commonly used in diabetes patients or may affect cancer risk (angiotensin converting enzyme inhibitor/angiotensin receptor blocker, calcium channel blocker, statin, fibrate, and aspirin). The living region and occupation were classified as detailed elsewhere (21). In brief, the living region was classified as Taipei, Northern, Central, Southern, and Kao-Ping/Eastern. Occupation was classified as class I (civil servants, teachers, employees of governmental or private businesses, professionals and technicians), class II (people without a specific employer, self-employed people or seamen), class III (farmers or fishermen), and class IV (low-income families supported by social welfare, or veterans). The ICD-9-CM codes for the related diagnoses are provided below: hypertension (401–405), dyslipidemia (272.0–272.4), obesity (278), nephropathy (580–589), eye disease (250.5: diabetes with ophthalmic manifestations, 362.0: diabetic retinopathy, 369: blindness and low vision, 366.41: diabetic cataract, and 365.44: glaucoma associated with systemic syndromes), stroke (430–438), ischemic heart disease (410–414), peripheral arterial disease (250.7, 785.4, 443.81, and 440–448), chronic obstructive pulmonary disease (a surrogate for smoking; 490–496), tobacco abuse (305.1, 649.0, and 989.84), alcohol-related diagnoses (291, 303, 535.3, 571.0–571.3, and 980.0), gallstone (574.00, 574.01, 574.10, 574.11, 574.20, 574.21, and A348), diagnoses related to Epstein-Barr virus infection (075, 710.3, and 710.4), hepatitis B virus infection (070.22, 070.23, 070.32, 070.33, and V02.61), hepatitis C virus infection (070.41, 070.44, 070.51, 070.54, and V02.62), and disease of pancreas (577). History of Helicobacter pylori infection was defined based on one of the following two criteria (22): 1) having received an eradication therapy for Helicobacter pylori (defined as a combination use of proton pump inhibitors or H2 receptor blockers, plus clarithromycin, metronidazole or levofloxacin, plus amoxicillin or tetracycline, with or without bismuth, in the same prescription order for 7–14 days); and/or 2) Helicobacter pylori infection diagnosis (041.86).

Analyses were conducted in both the unmatched original cohort and the matched cohort. The difference in age between never and ever users was compared by Student’s t test and Chi-square test was used to compare the differences of other variables. Standardized difference proposed by Austin and Stuart was calculated for each covariate and a value >10% was considered as potential confounding from the variable (23).

Incidence densities for never users, ever users and each tertile of cumulative duration of metformin therapy were calculated. The numerator was the case number of new-onset BTC identified during follow-up. The denominator was the person-years of follow-up, which ended at the time of BTC diagnosis or on the date of death, the last reimbursement record or December 31, 2011.

Kaplan-Meier curves for BTC-free probability for ever versus never users of metformin in the unmatched cohort and the matched cohort were plotted. Log-rank test was used to test the difference between ever and never users.

In main analyses, hazard ratios and their 95% confidence intervals for different subgroups of metformin exposure versus never users were created by three Cox regression models: 1) adjusted for PS; 2) incorporated with inverse probability of treatment weighting (IPTW) using the PS [as recommended by Austin to reduce confounding from the differences in characteristics (24)]; and 3) treating all covariates in **Table 1** as independent variables (traditional Cox model).

The following two sensitivity analyses were then conducted in the unmatched cohort. First, patients who happened to be treated with incretin-based therapies during follow-up were excluded. Incretin-based therapies including the dipeptidyl peptidase 4 inhibitors and the glucagon-like peptide 1 receptor agonists were introduced into Taiwan after the enrollment of the patients. On March 1, 2009 sitagliptin was the first incretin-based therapy approved for reimbursement by the NHI and it has been shown to reduce the risk of breast cancer in our population (25). Second, patients who developed cancers other than BTC during follow-up were excluded. These patients were excluded because the occurrence of other cancers might have shortened the lifespan of the patients leading to biased estimates of follow-up time. Furthermore, patients who developed other cancers might have different propensity for the risk of developing BTC.

To examine whether there might be interactions between metformin use and other potential confounders, multivariate traditional Cox regression models were created by entering metformin and all variables listed in **Table 1** (age divided into two subgroups: < 65 and ≥65 years) as independent variables together with the interaction term of metformin and each of the variables one at a time for estimating the *P* value of interaction. Because it was not known whether the follow-up duration might modify the effect, a similar model was created by adding follow-up duration (divided into two subgroups: <5 years and ≥5 years) as an additional independent variable together with the interaction term of metformin and follow-up duration for the estimation of the *P*-interaction.

To investigate whether the prognosis among incident cases of BTC might be different between ever and never users of metformin, the overall survival curves comparing ever versus never users were plotted for the unmatched cohort and the matched cohort, respectively. Log-rank test was used to test whether overall survivals could be significantly different between ever and never users.

SAS statistical software (version 9.4, SAS Institute, Cary, NC) was used for statistical analyses. *P* < 0.05 was considered statistically significant.

## Results

**Table 1** compares the characteristics between never and ever users of metformin. In the unmatched original cohort, age and sex differed significantly. The mean age was older (63.63 ± 10.42 versus 61.39 ± 10.22 years, *P*<0.0001) and the proportion of men was higher (57.31% versus 53.87%, *P*<0.0001) in never users. All other variables, except hypertension, pioglitazone, Epstein-Barr virus-related diagnoses and hepatitis B virus infection, also differed significantly in the unmatched original cohort. However, in the matched cohort, except for age, obesity, eye disease, insulin and sulfonylurea, all other variables were not different significantly. None of the standardized differences in the matched cohort had a value >10%, suggesting that the two groups were well matched.

**Figure 2:**
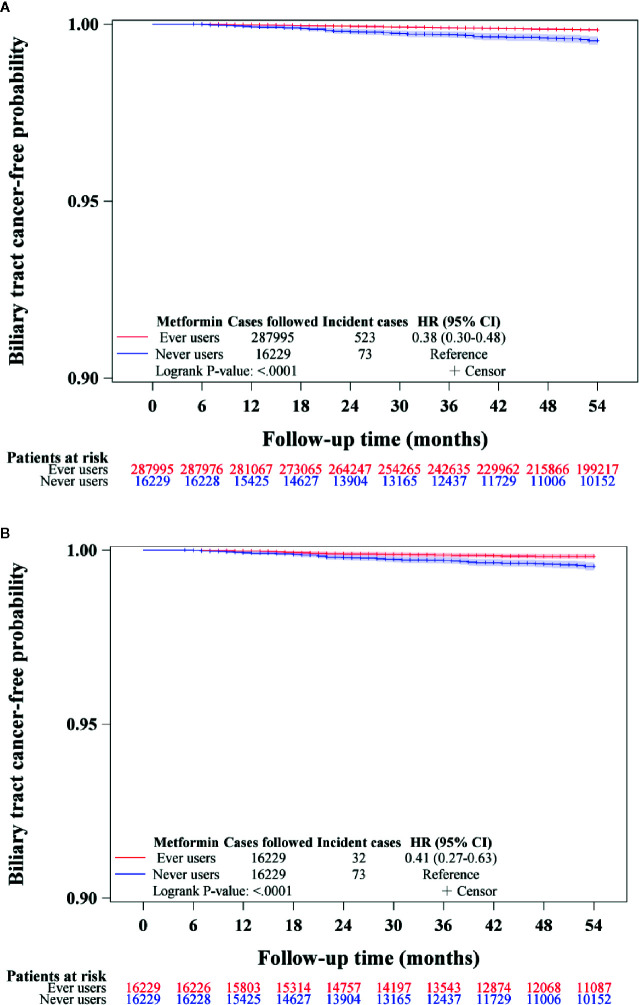
Kaplan-Meier curves for biliary tract cancer-free probability in never and ever users of metformin in the unmatched cohort **(A)** and matched cohort **(B)**. HR, hazard ratio; CI, confidence interval.

The Kaplan-Meier curves for BTC-free probability comparing ever versus never users of metformin are shown in **Figure 2**. The log-rank test suggested that ever users had a significantly lower risk of BTC by approximately 60% in either the unmatched cohort (**Figure 2A**)**** or the matched cohort (**Figure 2B**)****.

The incidence of BTC and the hazard ratios by metformin exposure in the main analyses are shown in **Table 2**. The overall hazard ratios suggested a significant reduction of BTC risk by approximately 50%–60% in most models. In the tertile analyses, the risk was neutral when metformin was used within a short period of approximately 2 years in the first tertile in all models. However, a significantly lower risk of BTC could be seen in the second and third tertiles in all models when metformin had been used for approximately 2 or more years. There was a trend of decreasing risk of BTC with increasing cumulative duration of metformin therapy.

**Table 2:** Incidence rates of biliary tract cancer and hazard ratios by metformin exposure in the main analyses.

Metformin use	*n*	*N*	Person-years	Incidence rate	PS-adjusted model			IPTW model			Traditional Cox model		
				(per 100,000 person-years)	HR	95% CI	*P* value	HR	95% CI	*P* value	HR	95% CI	*P* value
I. Unmatched original cohort													
Never users	73	16229	72739.47	100.36	1.000			1.000			1.000		
Ever users	523	287995	1374151.62	38.06	0.442	(0.344–0.568)	<0.0001	0.377	(0.295–0.481)	<0.0001	0.477	(0.370–0.615)	<0.0001
Tertiles of cumulative duration of metformin therapy (months)													
Never users	73	16229	72739.47	100.36	1.000			1.000			1.000		
<21.47	308	95194	344801.10	89.33	1.099	(0.847–1.426)	0.4756	0.889	(0.688–1.149)	0.3704	1.206	(0.926–1.572)	0.1643
21.47–46.00	160	94851	472510.95	33.86	0.388	(0.293–0.515)	<0.0001	0.331	(0.251–0.436)	<0.0001	0.430	(0.323–0.571)	<0.0001
>46.00	55	97950	556839.57	9.88	0.108	(0.075–0.153)	<0.0001	0.089	(0.063–0.127)	<0.0001	0.117	(0.082–0.167)	<0.0001
II. Matched cohort													
Never users	73	16229	72739.47	100.36	1.000			1.000			1.000		
Ever users	32	16229	77018.08	41.55	0.409	(0.270–0.619)	<0.0001	0.414	(0.273–0.627)	<0.0001	0.388	(0.254–0.593)	<0.0001
Tertiles of cumulative duration of metformin therapy (months)													
Never users	73	16229	72739.47	100.36	1.000			1.000			1.000		
<21.00	20	5346	18953.08	105.52	1.083	(0.659–1.780)	0.7544	1.054	(0.641–1.732)	0.8355	0.985	(0.589–1.648)	0.9542
21.00–46.07	10	5376	26762.41	37.37	0.368	(0.190–0.712)	0.0030	0.370	(0.191–0.717)	0.0032	0.356	(0.183–0.691)	0.0023
>46.07	2	5507	31302.59	6.39	0.061	(0.015–0.249)	<0.0001	0.062	(0.015–0.255)	0.0001	0.062	(0.015–0.252)	0.0001

n, new cases of biliary tract cancer during follow-up; N: cases followed; HR, hazard ratio; CI, confidence interval; PS, propensity score; IPTW, inverse probability of treatment weighting.

**Table 3** shows the incidence rates and hazard ratios in the sensitivity analyses after excluding patients who happened to be treated with incretin-based therapies and patients who developed other cancers during follow-up, respectively. The findings were consistent with the main analyses shown in **Table 2**, suggesting a significantly lower risk of BTC associated with metformin use in a dose-response pattern.

**Table 3:** Sensitivity analyses in the unmatched cohort.

Metformin use	*n*	*N*	Person-years	Incidence rate	PS-adjusted model			IPTW model			Traditional Cox model		
				(per 100,000 person-years)	HR	95% CI	*P* value	HR	95% CI	*P* value	HR	95% CI	*P* value
I. After excluding patients treated with incretin-based therapies during follow-up													
Never users	73	15245	68093.25	107.21	1.000			1.000			1.000		
Ever users	477	222884	1039481.45	45.89	0.488	(0.379–0.627)	<0.0001	0.426	(0.333–0.545)	<0.0001	0.528	(0.409–0.681)	<0.0001
Tertiles of cumulative duration of metformin therapy (months)													
Never users	73	15245	68093.25	107.21	1.000			1.000			1.000		
<21.47	293	81704	292242.24	100.26	1.125	(0.866–1.460)	0.3787	0.931	(0.719–1.204)	0.5838	1.248	(0.956–1.629)	0.1037
21.47–46.00	145	71223	350438.09	41.38	0.436	(0.327–0.580)	<0.0001	0.379	(0.286–0.502)	<0.0001	0.478	(0.358–0.639)	<0.0001
>46.00	39	69957	396801.12	9.83	0.099	(0.067–0.147)	<0.0001	0.084	(0.057–0.125)	<0.0001	0.108	(0.073–0.161)	<0.0001
II. After excluding patients who developed other cancers during follow-up													
Never users	73	14776	66710.33	109.43	1.000			1.000			1.000		
Ever users	523	267688	1278396.50	40.91	0.438	(0.341–0.563)	<0.0001	0.478	(0.371–0.616)	<0.0001	0.372	(0.291–0.475)	<0.0001
Tertiles of cumulative duration of metformin therapy (months)													
Never users	73	14776	66710.33	109.43	1.000			1.000			1.000		
<21.47	308	88142	318916.76	96.58	1.101	(0.849–1.428)	0.4683	1.220	(0.937–1.590)	0.1398	0.882	(0.682–1.139)	0.3356
21.47–46.00	160	87827	437962.13	36.53	0.388	(0.293–0.514)	<0.0001	0.435	(0.327–0.577)	<0.0001	0.328	(0.249–0.433)	<0.0001
>46.00	55	91719	521517.62	10.55	0.106	(0.075–0.151)	<0.0001	0.116	(0.082–0.166)	<0.0001	0.088	(0.062–0.125)	<0.0001

n: new cases of biliary tract cancer during follow-up; N: cases followed; HR, hazard ratio; CI, confidence interval; PS, propensity score; IPTW, inverse probability of treatment weighting.

Subgroup analyses and the *P* values of interaction are shown in **Table 4**. Except for Helicobacter pylori infection, no significant interaction between metformin use and any of the other variables was observed. For patients with a history of Helicobacter pylori infection, the protective effect of metformin was attenuated and not significant.

**Table 4:** Subgroup analyses and *P*-values for the interactions between metformin and each of the variables.

Subgroup	*n*	*N*	Person-years	Incidence rate (per 100,000 person-years)	Hazard ratio	95% Confidence interval	*P* value	*P*-interaction
Age								
<65 years	259	188107	888483.85	29.15	0.413	(0.277–0.618)	<0.0001	0.3299
≥65 years	337	116117	558407.23	60.35	0.517	(0.373–0.717)	<0.0001	
Follow–up duration								
<5 years	516	116553	365517.16	141.17	0.466	(0.356–0.610)	<0.0001	0.5496
≥5 years	80	187671	1081373.92	7.40	0.629	(0.296–1.335)	0.2270	
Sex								
Men	341	164442	774043.24	44.05	0.476	(0.342–0.663)	<0.0001	0.7416
Women	255	139782	672847.84	37.90	0.476	(0.320–0.707)	0.0002	
Occupation								
I	214	122502	584208.14	36.63	0.430	(0.284–0.649)	<0.0001	0.3867
II	136	69188	331327.95	41.05	0.456	(0.262–0.795)	0.0057	
III	153	59576	284360.84	53.80	0.480	(0.298–0.774)	0.0026	
IV	93	52958	246994.15	37.65	0.677	(0.332–1.381)	0.2835	
Living region								
Taipei	199	102734	489669.33	40.64	0.531	(0.335–0.841)	0.0070	0.2773
Northern	53	36090	172629.40	30.70	0.351	(0.144–0.856)	0.0213	
Central	109	54155	258985.61	42.09	1.129	(0.489–2.607)	0.7770	
Southern	117	48986	231224.18	50.60	0.386	(0.231–0.646)	0.0003	
Kao-Ping and Eastern	118	62259	294382.57	40.08	0.362	(0.217–0.602)	<0.0001	
Hypertension								
No	91	56142	260521.20	34.93	0.371	(0.210–0.656)	0.0007	0.1259
Yes	505	248082	1186369.89	42.57	0.508	(0.382–0.676)	<0.0001	
Dyslipidemia								
No	175	53404	242768.33	72.09	0.451	(0.300–0.678)	0.0001	0.6518
Yes	421	250820	1204122.75	34.96	0.497	(0.358–0.689)	<0.0001	
Obesity								
No	574	287293	1365567.67	42.03	0.477	(0.369–0.615)	<0.0001	0.7576
Yes	22	16931	81323.41	27.05	0.508	(0.060–4.266)	0.5328	
Nephropathy								
No	426	221371	1054889.26	40.38	0.415	(0.309–0.558)	<0.0001	0.1092
Yes	170	82853	392001.82	43.37	0.663	(0.403–1.088)	0.1041	
Eye disease								
No	438	212174	983621.65	44.53	0.420	(0.319–0.553)	<0.0001	0.2080
Yes	158	92050	463269.43	34.11	0.787	(0.381–1.625)	0.5166	
Stroke								
No	416	215496	1027548.42	40.48	0.432	(0.321–0.581)	<0.0001	0.1676
Yes	180	88728	419342.66	42.92	0.610	(0.372–1.000)	0.0500	
Ischemic heart disease								
No	291	165673	784975.25	37.07	0.405	(0.288–0.569)	<0.0001	0.1084
Yes	305	138551	661915.83	46.08	0.580	(0.395–0.851)	0.0054	
Peripheral arterial disease								
No	439	228313	1080230.64	40.64	0.457	(0.343–0.609)	<0.0001	0.5162
Yes	157	75911	366660.44	42.82	0.545	(0.315–0.942)	0.0297	
Insulin								
No	589	296778	1414845.00	41.63	0.474	(0.367–0.613)	<0.0001	0.5285
Yes	7	7446	32046.08	21.84	0.542	(0.058–5.074)	0.5911	
Sulfonylurea								
No	201	102653	455358.86	44.14	0.659	(0.350–1.241)	0.1966	0.1375
Yes	395	201571	991532.22	39.84	0.424	(0.319–0.563)	<0.0001	
Meglitinide								
No	570	292536	1390044.85	41.01	0.468	(0.360–0.609)	<0.0001	0.5612
Yes	26	11688	56846.23	45.74	0.621	(0.220–1.749)	0.3669	
Acarbose								
No	552	287863	1366149.24	40.41	0.458	(0.349–0.601)	<0.0001	0.4965
Yes	44	16361	80741.84	54.49	0.524	(0.226–1.215)	0.1318	
Rosiglitazone								
No	565	290788	1376190.67	41.06	0.477	(0.368–0.620)	<0.0001	0.9856
Yes	31	13436	70700.41	43.85	0.266	(0.072–0.984)	0.0472	
Pioglitazone								
No	582	296810	1411305.90	41.24	0.467	(0.362–0.604)	<0.0001	0.4526
Yes	14	7414	35585.18	39.34	0.698	(0.068–7.143)	0.7617	
Chronic obstructive pulmonary disease								
No	275	155573	739886.54	37.17	0.484	(0.333–0.706)	0.0002	0.9786
Yes	321	148651	707004.54	45.40	0.465	(0.330–0.657)	<0.0001	
Tobacco abuse								
No	573	292428	1390262.47	41.22	0.471	(0.365–0.609)	<0.0001	0.3794
Yes	23	11796	56628.61	40.62	0.719	(0.088–5.887)	0.7588	
Alcohol-related diagnoses								
No	524	282746	1350632.07	38.80	0.459	(0.352–0.600)	<0.0001	0.4066
Yes	72	21478	96259.01	74.80	0.672	(0.295–1.530)	0.3434	
Gallstone								
No	392	265783	1264864.56	30.99	0.483	(0.355–0.659)	<0.0001	0.5948
Yes	204	38441	182026.52	112.07	0.481	(0.309–0.749)	0.0012	
History of Helicobacter pylori infection								
No	308	212169	1012469.18	30.42	0.336	(0.244–0.463)	<0.0001	0.0021
Yes	288	92055	434421.90	66.30	0.740	(0.485–1.131)	0.1645	
Epstein-Barr virus infection								
No	588	302050	1436605.45	40.93	0.484	(0.375–0.626)	<0.0001	0.2249
Yes	8	2174	10285.63	77.78	0.167	(0.012–2.273)	0.1793	
Hepatitis B virus infection								
No	510	291423	1387187.11	36.77	0.514	(0.389–0.679)	<0.0001	0.3823
Yes	86	12801	59703.97	144.04	0.322	(0.173–0.599)	0.0003	
Hepatitis C virus infection								
No	503	288444	1373860.85	36.61	0.499	(0.376–0.662)	<0.0001	0.5089
Yes	93	15780	73030.23	127.34	0.392	(0.218–0.704)	0.0017	
Disease of pancreas								
No	519	289629	1379714.26	37.62	0.462	(0.353–0.605)	<0.0001	0.5743
Yes	77	14595	67176.82	114.62	0.613	(0.282–1.335)	0.2181	
Angiotensin converting enzyme inhibitor/angiotensin receptor blocker								
No	163	83691	384214.20	42.42	0.403	(0.264–0.617)	<0.0001	0.1896
Yes	433	220533	1062676.88	40.75	0.520	(0.378–0.715)	<0.0001	
Calcium channel blocker								
No	198	123486	581714.62	34.04	0.522	(0.338–0.807)	0.0035	0.7671
Yes	398	180738	865176.47	46.00	0.456	(0.333–0.623)	<0.0001	
Statin								
No	293	106328	486767.41	60.19	0.393	(0.286–0.538)	<0.0001	0.0531
Yes	303	197896	960123.68	31.56	0.668	(0.428–1.041)	0.0746	
Fibrate								
No	411	175795	822067.32	50.00	0.522	(0.386–0.707)	<0.0001	0.2215
Yes	185	128429	624823.76	29.61	0.381	(0.239–0.606)	<0.0001	
Aspirin								
No	228	119256	556831.57	40.95	0.376	(0.261–0.541)	<0.0001	0.0822
Yes	368	184968	890059.51	41.35	0.581	(0.406–0.832)	0.0030	

n: new cases of biliary tract cancer during follow-up; N: cases followed.

**Figure 3:**
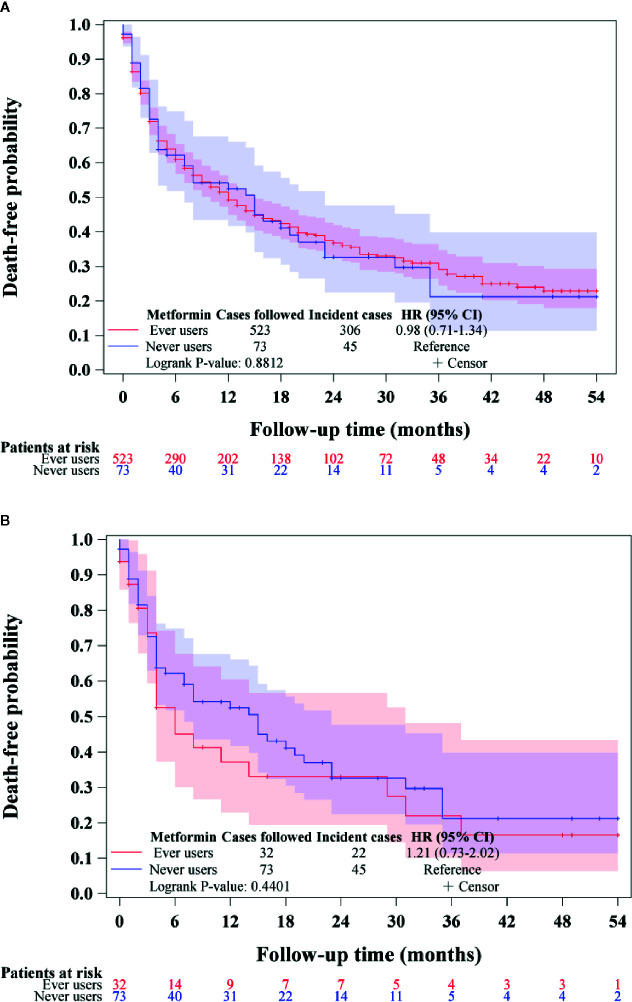
Overall survival in patients with incident biliary tract cancer in ever users and never users of metformin in the unmatched cohort **(A)** and the matched cohort **(B)**. HR, hazard ratio; CI, confidence interval.

**Figure 3** shows the overall survival in ever versus never users among those who had incident BTC in the unmatched cohort (**Figure 3A**) and the matched cohort (**Figure 3B**), respectively. The findings suggested that metformin did not affect the overall survival in patients with BTC after the diagnosis of the cancer.

## Discussion

The findings supported that metformin use in patients with type 2 diabetes mellitus was associated with a significantly lower risk of BTC in a dose-response pattern, which could be demonstrated in different regression models in either the main analyses (**Table 2**) or the sensitivity analyses (**Table 3**). Except for Helicobacter pylori infection, no significant interaction was observed for any of the other potential confounders (**Table 4**). Although metformin use was associated with a lower risk of BTC (**Figure 2**, **Tables 2** **–** **4**), the prognosis did not differ significantly between ever and never users once the patients developed BTC (**Figure 3**).

In our previous studies, metformin use was also associated with a lower risk of other types of cancer and the overall hazard ratios in well-matched cohorts were 0.72 (0.58–0.88) for lung cancer (19), 0.62 (0.53–0.74) for colorectal cancer (20), and 0.52 (0.31–0.89) for cervical cancer (26). Here the lowest overall hazard ratio of 0.414 (0.273–0.627) for BTC was observed in the corresponding Cox regression model incorporated with IPTW using the PS in the matched cohort (**Table 2**). Although metformin provided the most effective prevention on BTC, it might not be a useful therapeutic agent because its use was not associated with a better prognosis among incident cases of BTC (**Figure 3**). This is compatible with most previous studies that consistently showed a null effect when metformin was used for the treatment of BTC (10–12). Because BTC is highly malignant (1, 2), the more remarkable effect of metformin on the prevention of BTC renders a chance to reduce a greater burden of this life-threatening cancer if metformin is used for early prevention. A confirmation of such a chemopreventive effect of metformin on BTC by clinical trials, especially in high risk people, is urgently needed.

The protective effect of metformin on BTC was well demonstrated in the matched cohort (**Table 2**). The difference in age was small and slightly older in ever users in the matched cohort (**Table 1**). This might only have underestimated the beneficial effect of metformin because older age can be a risk factor of BTC. The slightly higher prevalence of obesity (2.71% vs. 2.15%), eye disease (18.55% vs. 17.15%) and insulin use (8.32% vs. 6.22%) in never users and the slightly higher prevalence of sulfonylurea use (75.43% vs. 72.69%) in ever users in the matched cohort were unlikely to cause significant residual confounding because all of their standardized differences were <10%.

The mechanisms explaining a reduced risk of BTC associated with metformin use may be multifactorial. The development and proliferation of BTC is favored by an alteration of cellular metabolism from oxidative phosphorylation to glycolysis (the Warburg effect) (3). The *in vitro* study by Tang et al. suggested that metformin may alter cholangiocarcinoma cancer cell metabolism and reverse the Warburg effect by reducing the expression of lactate dehydrogenase A (16). Metformin inhibits the mammalian target of rapamycin (mTOR) through an AMPK-dependent or an AMPK-independent pathway (27) and upregulation of mTOR is always observed in BTC (28). Metformin may also reduce inflammation, another feature of BTC (28), through the improvement of metabolic disturbances such as hyperglycemia, insulin resistance and dyslipidemia (29, 30). Transforming growth factor beta 1 plays an important role in the initiation and growth of BTC (31) and metformin has been identified as a suppressor of this growth factor in an *in vitro* study (32). Inactivation of tumor suppression function of FoxO3 is related to human development of BTC (33) and metformin activates the AMPK-FoxO3 pathway resulting in a reduction of intracellular reactive oxygen species (34). Lysophosphatidylcholine may cause cholangiocyte senescence which is potentially related to the development of BTC (35). It is interesting that metformin reduces lysophosphatidylcholine levels in human hepatocytes, which is related to the reduced secretion of Apo B (36).

Whether the anticancer effects of metformin on BTC may share similar mechanisms with many other types of cancer remains to be explored. However, because metformin has been consistently shown to reduce the risk of various types of cancer in the Taiwanese population, including cancers of the gastrointestinal system (20, 21, 37–40), gynecology-related cancers (26, 41–43), prostate cancer (44), cancers of the urinary system (45, 46), thyroid cancer (47), nasopharyngeal cancer (48), lung cancer (19), skin cancer (49), and non-Hodgkin lymphoma (50), it is possible that the anticancer effects of metformin may involve some common pathophysiological mechanisms relating to the development of various cancers. Hanahan and Weinberg pointed out six common hallmarks of cancer in 2000, including “sustaining proliferative signaling, evading growth suppressors, resisting cell death, enabling replicative immortality, inducing angiogenesis, and activating invasion and metastasis” (51). In 2011, they added two emerging hallmarks to the list, i.e., “reprogramming of energy metabolism and evading immune destruction” (52). It is interesting that metformin does show multi-faceted effects targeting most of these common cancer hallmarks relating to cancer development, proliferation and metastasis (52). Specifically, metformin inhibits cancer stem cells formation, inhibits epithelial-to-mesenchymal transition which is associated with cancer metastasis, influences the expressions of many microRNAs that may exert epigenetic effects on cancer development (53, 54), blocks the Warburg effect in energy metabolism that usually exists in cancer cells (53) and inhibits cellular senescence (53). Activation of mTOR is commonly observed in many types of cancer cells (51) and metformin is well recognized for its effects on the activation of AMPK, followed by the inhibition of mTOR (27).

The overall 50%–60% risk reduction in the present study (**Tables 2** and **3**) was comparable to that observed by Chaiteerakij et al. who used a clinic/hospital based case-control design to evaluate the risk of intrahepatic cholangiocarcinoma associated with metformin use (5). The investigators enrolled 612 cases of intrahepatic cholangiocarcinoma who were seen at the Mayo Clinic, Rochester, MN in the USA and 594 controls matched on age, sex, ethnicity, and residential area selected from participants in the Mayo Clinic Biobank. The effect of metformin was analyzed in the subgroup with diabetes mellitus. Because of its case-control design, only odds ratios could be estimated and the study did not evaluate a dose-response relationship. Additionally, the methodological problems associated with pharmacoepidemiological studies such as prevalent user bias, immortal time bias and confounding by indication were not addressed.

Basically, the present study has carefully addressed the limitations observed in the early study (5) by showing a dose-response relationship in various regression models (**Tables 2** and **3**). The potential risk of prevalent user bias, immortal time bias and confounding by indication have all been fully considered and will be discussed below.

Prevalent user bias can be introduced when prevalent users of a drug are enrolled to investigate its association with a certain clinical outcome (55). This bias has been avoided in the present study by enrolling patients with new-onset type 2 diabetes mellitus and new users of metformin (55).

Immortal time refers to the follow-up period when the researched outcome cannot happen (56). When treatment status and follow-up time are inappropriately assigned to the patients, immortal time bias can be introduced (56). In the present study, the diagnosis of diabetes mellitus and the assignment of treatment status would not be erroneous because only patients who had been diagnosed as having diabetes together with the prescription of antidiabetic drugs for 2 or more times were enrolled (**Figure 1**). Because the NHI is a universal healthcare system and it keeps all longitudinal information of the patients, never users without any prescription of metformin during the whole study period could also be easily and accurately identified. The immortal time during the period between diabetes diagnosis and the use of antidiabetic drugs were not included in the calculation of the follow-up time and the inappropriate assignment of follow-up time during the initial period of antidiabetic treatment had been avoided by excluding patients with a short follow-up duration of <180 days (**Figure 1**). Lévesque et al. discussed another source of potential immortal time bias that could be introduced during the wait period for getting the prescribed drugs when the patients were discharged from the hospital (56). It should be stressed that this would not happen in Taiwan because all prescribed drugs at discharge can be obtained directly and immediately from the hospital on the date when the patients are discharged.

Confounding by indication was less likely in the matched cohort with balanced confounders as indicated by all values of standardized difference <10% (**Table 1**). The use of Cox regression incorporated with IPTW using the PS was also aimed at reducing such a potential confounding by indication. Consistent findings in all regression models (**Tables 2** **–** **4**) strengthened the beneficial effect of metformin on BTC risk.

This study has several strengths. First, because of the high coverage rate, large sample size and nationwide basis of the NHI reimbursement database, the findings can be readily generalized to the whole population. Second, the inclusion of the unmatched cohort and matched cohort and the consistency in the findings across different methodological approaches in both study cohorts supported the robustness of the findings. Third, the use of medical records can reduce the potential bias related to self-reporting. Fourth, detection bias resulting from disparity in healthcare accessibility and socioeconomic status are less likely in Taiwan because the NHI is a compulsory and universal healthcare system with very low drug cost-sharing and most copayments can be waived in patients with cancer.

Study limitations may include the lack of measurement data on some potential risk factors such as anthropometric factors, smoking, alcohol drinking, lifestyle, physical activity, nutritional status, eating habits (such as raw or uncooked food), family history, and genetic markers. Because of lack of clinico-pathological features/parameters, the present study could not evaluate the impacts of the pathology, grading and staging of BTC. Because this study is retrospective in nature, further confirmation by prospective study or clinical trials is warranted. Finally, the findings observed in the diabetes patients should not be generalized to the nondiabetic people without additional confirmation.

In summary, this study supports a beneficial effect of metformin on the prevention of BTC in patients with type 2 diabetes mellitus in Taiwan. However, metformin may not affect the survival in patients with BTC. Because metformin is a cheap antidiabetic drug that is commonly used in clinical practice with few contraindications and without severe side effects, its beneficial effect on the prevention of a highly malignant cancer such as BTC in either the diabetes patients or the nondiabetic people is worthy of additional confirmation by clinical trials.

## Data Availability Statement

The datasets for this article are not publicly available because public availability of the dataset is restricted by local regulations to protect privacy. Requests to access the datasets should be directed to ccktsh@ms6.hinet.net.

## Ethics Statement

The studies involving human participants were reviewed and approved by National Health Research Institutes. Written informed consent for participation was not required for this study in accordance with the national legislation and the institutional requirements.

## Author Contributions 

The author confirms being the sole contributor of this work and has approved it for publication.

## Funding

The study was supported by the Ministry of Science and Technology (MOST 107-2221-E-002-129-MY3) of Taiwan and the Yee Fong Charity Foundation. The funders had no role in study design, data collection and analysis, decision to publish, or preparation of the manuscript.

## Conflict of Interest

The author declares that the research was conducted in the absence of any commercial or financial relationships that could be construed as a potential conflict of interest.

## Abbreviations

BTC, biliary tract cancer; AMPK, 5’-adenosine monophosphate-activated protein kinase; PS, propensity score; NHI, National Health Insurance; ICD-9-CM, International Classification of Diseases, Ninth Revision, Clinical Modification; IPTW, inverse probability of treatment weighting; mTOR, mammalian target of rapamycin.
